# Insights into the kinetics and dynamics of the furin-cleaved form of PCSK9

**DOI:** 10.1194/jlr.RA120000964

**Published:** 2020-11-23

**Authors:** Carlota Oleaga, Joshua Hay, Emma Gurcan, Larry L. David, Paul A. Mueller, Hagai Tavori, Michael D. Shapiro, Nathalie Pamir, Sergio Fazio

**Affiliations:** 1 Knight Cardiovascular Institute, Center for Preventive Cardiology, Oregon Health & Science University, Portland, OR, USA; 2 Proteomics Shared Resource, Oregon Health & Science University, Portland, OR, USA

**Keywords:** cardiovascular disease, LDL cholesterol, lipoprotein metabolism, LDL receptor, proprotein convertases, posttranslational modifications, PCSK9, aa, amino acid, ER, endoplasmic reticulum, LDL-C, low density lipoprotein cholesterol, PCSK9, Proprotein convertase subtilisin/kexin type 9, ProPCSK9, precursor form of PCSK9, PCSK9_62, mature form of PCSK9, PCSK9_55, furin-cleaved form of PCSK9 (55 kDa), pFURIN, expression plasmid for furin, pPCSK9 62, expression plasmid for PCSK9_62, pPCSK9 ΔPD, expression plasmid for PCSK9_62 lacking the prodomain, pPCSK9 55, expression plasmid for PCSK9_55, pPCSK9 PD, expression plasmid for PCSK9 prodomain, pPCSK9 62∗R46L, expression plasmid for PCSK9_62 R46L, pPCSK9 62∗R218S, expression plasmid for PCSK9_62 R218S

## Abstract

Proprotein convertase subtilisin/kexin type 9 (PCSK9) regulates cholesterol metabolism by inducing the degradation of hepatic low density lipoprotein receptors (LDLRs). Plasma PCSK9 has 2 main molecular forms: a 62 kDa mature form (PCSK9_62) and a 55 kDa, furin-cleaved form (PCSK9_55). PCSK9_55 is considered less active than PCSK9_62 in degrading LDLRs. We aimed to identify the site of PCSK9_55 formation (intracellular vs. extracellular) and to further characterize the LDLR-degradative function of PCSK9_55 relative to PCSK9_62. Coexpressing PCSK9_62 with furin in cell culture induced formation of PCSK9_55, most of which was found in the extracellular space. Under the same conditions, we found that *i)* adding a cell-permeable furin inhibitor preferentially decreased the formation of PCSK9_55 extracellularly; *ii)* using pulse-chase analysis, we observed the formation of PCSK9_55 exclusively extracellularly in a time-dependent manner. A recombinant form of PCSK9_55 was efficiently produced but displayed impaired secretion that resulted in its intracellular trapping. However, the nonsecreted PCSK9_55 was able to induce degradation of LDLR, though with 50% lower efficiency than PCSK9_62. Collectively, our data show that *1)* PCSK9_55 is formed extracellularly; *2)* PCSK9_55 has a shorter half-life; *3)* there is a small intracellular pool of PCSK9_55 that is not secreted; and *4)* PCSK9_55 retained within the cell maintains a reduced efficiency to cause LDLR degradation.

Elevated low density lipoprotein (LDL) cholesterol (LDL-C) levels are a major risk factor for cardiovascular disease (CVD) (1, 2, 3). The LDL particle is cleared from circulation by the hepatic LDL receptor (LDLR), which recycles back to the membrane several hundred times in its 20 hour life span (4, 5). Proprotein convertase subtilisin/kexin type 9 (PCSK9) is a secreted protein that binds hepatic LDLR and prevents its recycling by chaperoning it to lysosomal degradation (6, 7, 8, 9, 10). PCSK9 plays a key role in lipoprotein metabolism (11, 12) and is a therapeutic target to lower plasma LDL-C levels in high-risk individuals (13, 14).

PCSK9 is mainly produced by the liver and undergoes several posttranslational modifications that may influence its kinetics and function (15, 16, 17, 18, 19, 20). Synthesized as a proprotein of 75 kDa (ProPCSK9), PCSK9 undergoes autocatalytic cleavage at the N-terminus after residue 152 while traveling through the secretory pathway. The released prodomain (aa 32–152, 13 kDa in length) remains attached to the N-terminal region of the protein by noncovalent forces generating a heterodimer (62 + 13 kDa). This heterodimer is the mature form of PCSK9 (PCSK9_62). Cleavage of the prodomain is essential for secretion of PCSK9_62 into circulation (9, 21). PCSK9_62 can also undergo a second proteolytic cleavage mediated by the protease furin. Furin cleaves PCSK9 at the N-terminal region, after residue 218, releasing a ∼7 kDa peptide and generating the second most common form of active plasma PCSK9 (PCSK9_55) with a size of 55 kDa (15, 22, 23). The mature and furin-cleaved forms are both present in circulation (15, 19), with 60%–75% as PCSK9_62 and 25%–40% as PCSK9_55 (24, 25, 26). It is worth noting that there are no commercially available methods to differentiate between PCSK9 forms (PCSK9_62 vs. PCSK9_55) in plasma (17, 27).

Furin is a ubiquitously expressed convertase capable of processing multiple protein targets in different cellular compartments (i.e., trans-Golgi network, endosomal compartment, cell surface, and the extracellular space) (28, 29, 30, 31, 32). Due to their cellular dynamics, furin and PCSK9 may come in contact with each other along the secretory pathway, on the cell membrane or in the extracellular space (15, 22). Benjannet *et al.* (15) identified the endoglycosidase H-resistant form of PCSK9_62 as the isoform most efficiently cleaved by furin, indirectly identifying the Golgi compartment as the site of PCSK9's cleavage by furin. In addition, the authors claimed that once furin cleaves PCSK9_62, newly formed PCSK9_55 would be rapidly secreted to the extracellular space, but they did not provide evidence in support of this claim (15). Later, Essalmani *et al.* (22) proposed a mechanism where the cleavage of PCSK9_62 occurs extracellularly and is mediated by the transmembrane form of furin. Thus, the site of PCSK9's cleavage by furin remains unclear.

Point mutations that affect the furin recognition site on PCSK9 (–Arg–X–Lys/Arg–Arg^↓^–) impair its proteolysis and act as gain-of-function (GOF) mutations, increasing LDLR degradation (e.g., the PCSK9-R218S mutant). These clinical observations first suggested that the furin-cleaved form of PCSK9 would have reduced functionality (33, 34). Furthermore, the hypolipidemic effects observed in carriers of mutations in angiopoietin-like 3 are associated with increased plasma concentration of PCSK9_55 (26). Accordingly, multiple in vitro studies have demonstrated that extracellular PCSK9_55 is inactive, or less active than PCSK9_62 in degrading LDLRs (15, 24, 25). While it is known that PCSK9_62 can induce LDLR degradation via both extracellular and intracellular pathways (35, 36), an intracellular pathway of LDLR degradation has not been identified for PCSK9_55.

In this study, we aimed to identify the site of furin-dependent PCSK9_55 formation and to further characterize its LDLR-degradative properties relative to PCSK9_62. Our results demonstrate that the coexpression of PCSK9_62 and furin leads to production of PCSK9_55 that is localized predominately in the extracellular space. Under the same conditions, the addition of a furin inhibitor with access to both intracellular and extracellular compartments selectively reduced the efficiency of cleavage only extracellularly. Our expression studies with engineered PCSK9_55 demonstrate that the intracellular pool of PCSK9_55 is not secreted to the extracellular space, suggesting that, under physiologic conditions, the intracellular PCSK9_55 originates from endocytosis of the plasma form, rather than from cleavage by intracellular furin. This was supported by a pulse-chase experiment that demonstrates PCSK9_55 first appears at the extracellular compartment. In the same experiment, we also confirmed that PCSK9_55 has a shorter half-life than PCSK9_62. We also determined that intracellular PCSK9_55 has the ability to induce the degradation of LDLRs, albeit with only 50% of the capacity compared with PCSK9_62. Taken together, our results suggest that *1)* PCSK9_55 is formed extracellularly; *2)* PCSK9_55 has a shorter half-life; *3)* there is a small intracellular pool of PCSK9_55 that is not secreted; and *4)* PCSK9_55 retained within the cell maintains a reduced efficiency to cause LDLR degradation.

## Materials and methods

### Materials

All products were purchased from Sigma-Aldrich unless otherwise noted.

### Cell culture

Human embryonic kidney HEK293T cells (ATCC® CRL-1573™) and hepatocellular carcinoma HepG2 cells (ATCC® HB-8065™) were routinely grown as previously described (37) at 37°C in a 5% CO_2_ humidified atmosphere. Composition of cell media is detailed in supplemental Table S1.

### Furin inhibition

Furin inhibition was achieved using cell-permeable Decanoyl-RVKR-CMK added to cell media at 25 and 50 μM (Cat# 344930, Millipore) (38).

### Plasmid DNA and transfections

pcDNA3 expression plasmids for human PCSK9_62 (pPCSK9 62) and PCSK9_62 lacking the prodomain (pPCSK9 ΔPD, removed amino acid 32–152) were previously developed in our laboratory (37). pcDNA3 expression plasmids for human PCSK9 lacking amino acids 32–218 (pPCSK9 55) and 153–692 (pPCSK9 PD) were generated by deleting specific sequences from pPCSK9 62 by inverse PCR using a high-fidelity polymerase (Cat# M0530, Phusion® HF, New England Biolabs) and the designed primers (supplemental Table S2). Linearized DNA products were purified (Cat# K0691, GeneJET, Thermo Scientific) and closed by ligation with a T4 ligase (Cat# M0202S, New England Biolabs) after phosphorylating the 5' ends with a T4 polynucleotide kinase (Cat# M0201S, New England Biolabs). Plasmid DNAs were transformed in bacteria, and glycerol stocks were stored at -80°C. Correct deletions were verified by Sanger sequencing. The plasmid pBa-LSS-GFP-LDLR wt that codifies for a green fluorescent protein (GFP)-tagged-hLDLR was provided by Addgene (Plasmid #98184), originated and deposited by Drs Banker and Bentley (39). pFURIN-pcDNA3 expression plasmid for human furin was a gift from Dr Shinde (pFURIN) (40). Expression plasmids for 2 natural mutants of PCSK9, *i)* R46L (pPCSK9 62∗R46L or pIR-hPCSK9 FL + v5 (R46L)), a PCSK9 LOF mutation correlated with 15% reduction in LDL-C and 47% reduction in coronary heart disease risk (12, 41), and *ii)* R218S (pPCSK9 62∗R218S or pIR-hPCSK9 FL + v5 (R218S)), a PCSK9 GOF mutation resistant to furin cleavage and correlated to higher risk for CVD (15, 33, 34), were kindly provided by Dr Seidah (15).

Transient transfection was performed following the manufacturer's instructions with a nonliposomal agent. Briefly, cells were plated (2.5 × 10^5^ cells per well) in 6-well plates; on day 2, media was changed to serum-free media (900 μL); and cells were transfected with the DNA-agent complex (100 μL/well of 4.5 μL of Fugene 6 (Cat# E269, Promega) per 1.5 μg of plasmid DNA in DMEM media). Serum was withdrawn until the end of the experiment (72 h) to avoid interference. Cells and conditioned media were collected on day 5 for protein evaluations with Western blot, ELISA, or flow cytometry. Cell extracts were obtained from cells washed twice with ice-cold phosphate-buffered saline and scraped in 100 μL of lysis buffer (RIPA buffer (Cat# R0278, Sigma-Aldrich) supplemented with 1X proteinase inhibitor (Cat# A32963, Thermo Fisher Scientific)). Lysates were incubated on ice for 1 h and vortexed every 15 min and then spun at 14,000 *g* at 4°C for 20 min. Collected supernatants were quantified by a Lowry assay (DC™ Protein Assay, Cat# 5000111, Bio-Rad) and then stored at −20°C until used.

### Western blot analyses

Proteins (protein extracts [40 μg], cell-conditioned media [20 μL], or purified proteins [150–400 ng]) were resolved in SDS-PAGE (4%–12% Bis-Tris precast acrylamide gels, Cat# NP0321BOX, Thermo Fisher Scientific) and later transferred to nitrocellulose membranes (Cat# 10600003, GE Healthcare) and blotted for human PCSK9, LDLR, furin, or actin (supplemental Table S3). Antibody dilutions were done in blocking buffer (Cat# 927-50003, LI-COR Biosciences). Incubations with primary antibodies were done overnight at 4°C and with secondary antibodies were done for 2 h at room temperature. Membranes were scanned with an Odyssey® CLx imaging system. Protein band size was estimated relative to the protein ladder (Chameleon® Duo Pre-stained Protein Ladder, Cat# 928-60000, LI-COR Biosciences).

Image Studio Lite Ver 5.2 software (LI-COR Biosciences) was used to quantify band intensity. PCSK9 band intensity from total extracts (40 μg/lane) was normalized to actin band intensity, whereas PCSK9 band intensity from cell-conditioned media (20 μL/lane [equivalent to 16.1 ± 0.6 μg of secreted proteins from cells maintained under serum-free conditions]) was not normalized. Quantification of PCSK9 intensities was done separately in cell extracts and in cell media compartments due to the irrationality of normalizing by total protein content. Total PCSK9 band intensity (PCSK9_62 + PCSK9_55 or ProPCSK9 + PCSK9_62 + PCSK9_55 + PCSK9 PD) found in pPCSK9 62 (±pcDNA3) transfected cells condition was considered as the reference, and other conditions were quantified relative to it. Note that there is an ∼8X difference between the relative volume loaded from the intracellular and the extracellular compartments in the gels. Cell extracts are loaded as ∼1/6.25 of the total volume of extracts (40 μg of total extracts corresponded 16 ± 2 μL out of the total ∼100 μL), while cell media is loaded as ∼1/50 of the total volume of media in the well (20 μL out of the total 1,000 μL).

### Removal of N-glycosylation radicals

Conditioned media (9 μL) and cell extracts (20 μg) of HEK293T cell were subjected to N-glycosylation removal with the amidase PNGase F that cleaves between the innermost GlcNAc and the asparagine residue of high mannoses, following vendor's instructions (Cat# CP0704S, New England Biolabs), and later subjected to Western blot as described above. HepG2 cells were treated with tunicamycin (5 μg/mL, added 16 h after transfection) (Cat# 11445, Cayman Chemical) to inhibit N-glycosylation, thus generating the nonglycosylated PCSK9 form.

Cell extracts (20 μg) of HEK293T cell were subjected to Endoglycosidase H (Endo H) digestion following vendor's instructions (Cat# P0702S, New England Biolabs) and later subjected to Western blot as described above.

### PCSK9 quantification by ELISA

Human PCSK9 was quantified in the cell extracts (25 μg of total protein) and cell-conditioned media (1:10–12.5 dilution) of HEK293T cells transfected with pPCSK9 62, using a human PCSK9 ELISA kit (Cat# DPC900, R&D Systems Quantikine) and following manufacturer's instructions. Total PCSK9 content in each compartment (intracellular and extracellular) was calculated based on the final volume of the cell extracts (100 μL) and cell media (1,000 μL).

### In vitro PCSK9 proteolysis mediated by furin

The reaction conditions were adapted from published work (25, 26). PCSK9 from different sources (cells overexpressing PCSK9 (3.4 μL of media or 3.4 μL of cell extract) or a purified recombinant form; 1900 nM, Cat# 20631, Cayman Chemical) was set to react with furin (680 nM; Cat# 450-47, PeproTech) under optimized buffer, temperature, and time conditions (4 mM CaCl_2_, 150 mM NaCl, 20 mM KCl and 50 mM Tris-HCl pH 7.4 in 10 μL of final volume at 37°C for 24 h) to achieve the highest yield of PCSK9 proteolysis. Reactions were stopped by chelating calcium with 4 mM EDTA. Products were analyzed by Western blot or mass spectrometry.

### Liquid chromatography-mass spectrometry of furin-cleaved PCSK9

The products obtained from in vitro PCSK9 proteolysis with furin were subjected to liquid chromatography-mass spectrometry (LC-MS). The designed method skipped the common protein digestion step used in MS, to enhance the detection of the released 7 kDa cleaved peptide in a qualitative manner, since it will only be expected in the furin-containing reaction. Two biological replicates including the products of a negative control reaction (no furin; PCSK9 62 kDa) and the experimental reaction PCSK9_62 with furin (PCSK9 55 kDa) were evaluated separately in 2 independent LC-MS analyses to detect the expected furin cleavage product containing residues 153–218 (average mass = 7730.5). With no further digestion, aliquots of 2.2 μg of PCSK9 from a control sample (no furin) and a furin-incubated sample were dried using a DNA120 SpeedVac (Thermo Scientific) and stored at −20°C until analysis. Samples were solubilized in 22 μL of 8 M urea for 24 h prior to analysis. A total of 1 μg of protein was analyzed using an LTQ Velos Pro linear ion trap (Thermo Scientific). Samples were injected onto a micro protein trap cartridge (Optimize Technologies) at a flow rate of 20 μl/min in mobile phase containing 0.1% formic acid. After 5 min, the flow was diverted to a 1 × 250 mm C4 column (Vydac, SN 214MS51, Grace). Protein was eluted by increasing the acetonitrile concentration from 2% to 7.5% over 1 min and then 7.5% to 60% over 30 min, and data collection on the mass spectrometer started 10 min into the separation. The instrument used an HESI-II probe fitted with a 34 gauge metal needle, 5.0 kV source voltage, 325°C ion-transfer tube temperature, sheath gas setting of 5, full MS scans in profile mode over a range of m/z = 400–2000, and averaging of 10 μscans. Spectra acquired during elution of the PCSK9 153–218 peaks were then averaged and deconvoluted using the Manual Respect module for isotopically unresolved data in Protein Deconvolution 4.0 software (Thermo Scientific). Single-ion chromatographic traces were produced using Qual Browser software within the Xcalibur Suite (Thermo Scientific). Peptide identification was unnecessary given the fact that we used purified PCSK9. Annotated mass spectra are available at PanoramaWeb (https://panoramaweb.org/kqwUPs.url), a server hosted by the Department of Genome Sciences at the University of Washington (42).

### Pulse-chase studies

On day 1, HEK293T cells were plated (2.5 × 10^5^ cells per well) in 6-well plates and maintained as detailed above. On day 2, cells were cotransfected with pPCSK9 62 + pcDNA3 or pPCSK9 62 + pFURIN as explained above. Pulse-chase experiments started on day 4 following the methods followed in (43, 44); all the media formulations used for this protocol are detailed in supplemental Table S4. Cells were initially starved from methionine and cysteine amino acids by removing the medium, rinsing the cells twice with 1X PBS, and incubating the cells with 1 mL of starvation media for 1 h. Then, cells were pulsed by replacing the media with 0.7 mL of labeling media for 30 min. Chasing was started by removing the labeling media, rinsing the cells twice with 1 mL of 1X PBS and adding 1 mL of chasing media. At the selected time points, 0, 1, 2, 5, and 22 h after addition of chasing media, cell media was collected and cells were rinsed twice with 1X PBS. Cell extracts were collected as explained above. PCSK9 was immunoprecipitated from the collected cell media and extracts using Dynabeads protein G immunoprecipitation (IP) kit (Cat #10007D, Thermo Fisher Scientific), following vendor's recommendations. In brief, 5 μg of rabbit polyclonal antibody against human PCSK9 (Cat# CY-1307, MBL international) was coupled to protein G-coated magnetic beads and cross-linked with 5 mM BS3 (Cat # 21580, Thermo Fisher Scientific). PCSK9 was pulled from samples (1 mL of conditioned media and 150 μg of total extracts) after incubation with antibody-bead complex, and PCSK9 was released from the complex under denaturing conditions. Samples were resolved in SDS-PAGE (4%–12% Bis-Tris precast acrylamide gels, Cat# NP0321BOX, Thermo Fisher Scientific). Gels were dried at 80°C for 4 h (Cat# 165-1745, Bio-Rad) and exposed to storage phosphor screen. Signals were obtained by scanning the screen with a phosphor imager (Typhoon Trio, Amersham Biosciences). Band intensity was quantified relative to the control at time 0 h in the cells extracts and time 1 h in cell media.

### PCSK9 functional assay

HEK293T cells were plated (2.5 × 10^5^ cells per well) in 6-well plates. The following day, media was changed to serum-free and cells were transfected to express GFP-tagged LDLR. Serum was then withdrawn until the end of the experiment (72 h) to avoid interference. Exposure to PCSK9 was introduced either by cotransfecting cells with a plasmid expressing PCSK9 on day 2 or by adding purified PCSK9_62 to the medium (3 μg/mL [Cat# 20631, Cayman Chemical]) on day 4. On day 5, cells were harvested, spun (300 *g*, 3 min, 4°C), and washed with 1 mL of flow buffer (25 mM Hepes, pH 7; 2 mM EDTA, pH 8; and 5.5 mM glucose in 1X PBS). Cells were spun again and resuspended with 0.5 mL of flow buffer containing 2.5 ng/μL propidium iodide (Cat# P4170, Sigma-Aldrich), used as a live/dead discriminator. Compensation was performed using unstained and single-stained cells. Acquisition was performed on BD FACSCanto™ II instrument using BD FACSDiva™ software, and analysis was performed using Flow Jo v10. The gating strategy followed to quantitatively monitor PCSK9 activity is detailed in supplemental Fig. S1.

### Endoplasmic reticulum stress evaluation

The mRNA expression levels of spliced X-box binding protein-1 (XBP-1s) was evaluated as a predictor of endoplasmic reticulum (ER) stress, following the method followed in (45). RNA was extracted from HEK293T cells on day 5 after cotransfections using E.Z.N.A.® Total RNA Kit I (Cat# R6834-02, OMEGA Bio-Tek). One microgram of RNA was used to generate cDNA by reverse transcription (iScript cDNA Synthesis Kit, Cat# 1708891, Bio-Rad). Fifty nanograms of cDNA template was used for the qRT-PCR reactions. *XBP-1s* and *RPL32* gene expressions were assayed via TaqMan technology with TaqMan probes (Hs03929085_g1 and Hs00851655_g, Thermo Fisher Scientific) and a TaqMan Master mix (TaqMan Universal Master Mix II, no UNG, Cat # 4440040, Thermo Fisher Scientific), following vendor's recommendations. Changes in gene expression were calculated using the quantitative ΔΔCt method, normalized against *RPL32*, and plotted relative to the control condition.

### Statistical methods

Values are expressed as mean ± SEM with a minimum of 3 independent experiments. Change between 2 conditions was evaluated by unpaired Student's *t*-test analysis run with a 2-tail distribution in Microsoft Excel Statistical tool or Prism 8.3 (GraphPad Software, LLC). Kinetic changes studied in the pulse-chase experiments were evaluated by 2-way ANOVA. Differences with *P*-values < 0.05 were considered statistically significant.

## Results

### PCSK9_55 is mainly located in the extracellular space

**Fig. 1:**
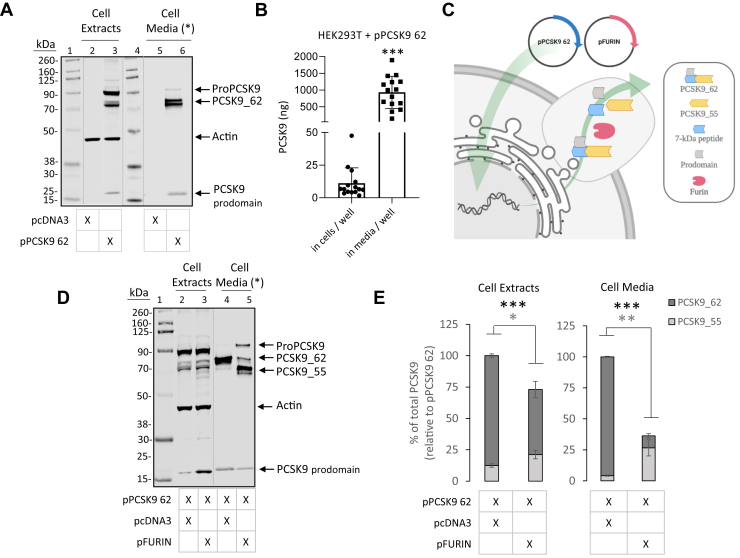
Generation of PCSK9_55 in cells culture. (A) Extracts and conditioned media of HEK293T cells transfected with empty vector (lanes 2 and 5) or with pPCSK9 62 (lanes 3 and 6) were separated via SDS-PAGE and immunoblotted for PCSK9 and actin (MW ladder; lanes 1 and 4). Image is representative of 3 independent experiments. (B) Total PCSK9 quantified in the intracellular and extracellular compartments of our in vitro conditions in HEK293T cells transfected with pPCSK9 62. (C) Experimental strategy followed in cell culture to generate PCSK9_55 under semiphysiologic conditions cotransfecting pPCSK9 62 and pFURIN. (D) Extracts and conditioned media from HEK293T cells transfected with pPCSK9 62 and empty vector (lanes 2 and 4) or pPCSK9 62 and pFURIN (lanes 3 and 5) were separated on an SDS-PAGE and immunoblotted for PCSK9 and actin (MW ladder; lane 1). (E) Quantification of PCSK9 bands from “D” are plotted relative to pPCSK9 62 in the cell extracts and cell media. Image is representative of ≥13 independent experiments. Values are mean ± SEM. ∗*P* < 0.05, ∗∗*P* < 0.01, and ∗∗∗*P* < 0.001. (∗) Cell media is loaded with unequal protein load as in cell extracts. Dotted lines indicate splicing of original image.

We induced expression of PCSK9_62 or PCSK9_55 by transient transfection in HEK293T cells lacking native PCSK9. Cotransfection with pPCSK9_62 and empty vector (pcDNA3) resulted in the expression and accumulation of ProPCSK9 and PCSK9_62 in the cell and secretion of PCSK9_62 into the media (Fig. 1A), as expected and as previously reported (15, 46). PCSK9_62 was found in its nonglycosylated and N-glycosylated forms, both intracellularly and in the media (supplemental Fig. S2A). Quantification of PCSK9 showed that the extracellular compartment contained 84-fold more PCSK9 than the intracellular compartment (Fig. 1B). Cotransfection of cells with pPCSK9_62 and pFURIN resulted in the generation of PCSK9_55 which increased moderately in the intracellular compartment (70 ± 27%; *P* < 0.05) and more robustly in the extracellular compartment (552 ± 185%; *P* < 0.001) (Fig. 1C–E). Overall, there was nearly an order of magnitude difference between the PCSK9_55 accumulated in the extracellular compartment (258 ng) and that accumulated intracellularly (2.7 ng; supplemental Table S5, extrapolated from data in Fig. 1B, E). Besides inducing the formation of PCSK9_55, furin decreased total PCSK9 (sum of PCSK9_62 and PCSK9_55) in both cellular compartments (intracellular, 32 ± 13% reduction *P* = 0.001; and extracellular, 75 ± 6% reduction *P* < 0.001). Furin also altered the PCSK9 prodomain levels, with a 146 ± 46% increase intracellularly (*P* < 0.01) and a 83.7 ± 2.6% reduction in the extracellular space (*P* < 0.001) (supplemental Fig. S3). We repeated these experiments using HepG2 cells and obtained similar results including the presence of PCSK9 in both nonglycosylated and glycosylated forms (supplemental Fig. S2B), and the formation of PCSK9_55 that accumulated predominately in the cell media after cotransfection of PCSK9_62 and furin (supplemental Fig. S4A, B).

Furin-specific cleavage was corroborated by reacting purified PCSK9 (nonglycosylated) with furin and analyzing reaction products both by Western blotting and mass spectrometry (supplemental Fig. S5) and by evaluating furin cleavage site-selectivity comparing PCSK9 WT, a common human variant called PCSK9 R46L (12, 41), and a furin-resistant variant called PCSK9 R218S (15, 33, 34) (supplemental Fig. S6).

Under these conditions, *i)* PCSK9_62 accumulated in the extracellular space with little intracellular retention; *ii)* both PCSK9_62 and PCSK9_55 expressed the nonglycosylated and N-glycosylated forms; *iii)* furin induced the formation of PCSK9_55 from PCSK9_62, and PCSK9_55 was mainly located extracellularly; and *iv)* furin cleavage of PCSK9_62 induced a decrease in total PCSK9 accumulation and changes in PCSK9 prodomain.

### Furin cleaves PCSK9_62 in the extracellular space

**Fig. 2:**
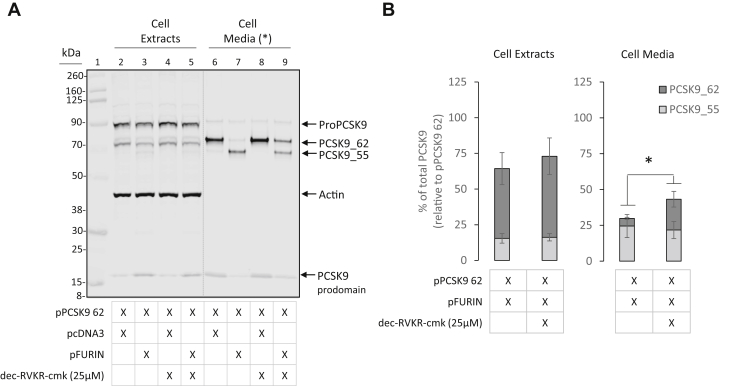
Furin inhibition selectively limits PCSK9_62 conversion to PCSK9_55 in the extracellular space. HEK293T cells cotransfected with pPCSK9 62 and pFURIN were treated with the cell-permeable furin inhibitor dec-RVKR-cmk (25 μM) to repress PCSK9 cleavage. (A) Extracts and conditioned media of HEK293T cells transfected with pPCSK9 62 and empty vector (lanes 2 and 6, and treated with furin inhibitor lanes 4 and 8) or pPCSK9 62 and pFURIN (lanes 3 and 7, and treated with furin inhibitor lanes 5 and 9) were separated via SDS-PAGE and immunoblotted for PCSK9 and actin (MW ladder; lane 1). (B) Quantification of PCSK9 bands from “A” are plotted relative to pPCSK9 62 in the cell extracts and cell media. Image is a representative of 4 independent experiments. Values are mean ± SEM. ∗*P* < 0.05. (∗) Cell media is loaded with unequal protein load as in cell extracts. Dotted lines indicate splicing of the original image.

To identify the furin pool responsible for PCSK9_55 generation, we inhibited furin activity in cell culture with decanoyl-RVKR-CMK, a cell-permeable peptide antagonist of furin (38). Briefly, HEK293T cells were cotransfected with pPCSK9 62 and pFURIN to generate PCSK9_55, as in Fig. 1C–E, and decanoyl-RVKR-CMK was added 8 h after transfection to block furin activity in selected conditions. Treatment with 25 μM decanoyl-RVKR-CMK reduced furin-dependent conversion of PCSK9_62 to PCSK9_55 in the extracellular compartment by 32 ± 4% (*P* = 0.0376) when compared with nontreated cells, while causing no significant changes in the intracellular compartment (Fig. 2A). The furin inhibitor preserved PCSK9_62 form in the extracellular compartment. There was 4-fold (*P* < 0.05) more PCSK9_62 in the media when furin inhibitor was added to the pPCSK9 62 and pFURIN cotransfection condition (Fig. 2B). Interestingly, the location for furin cleavage of PCSK9_62 appeared to be extracellular despite the fact that furin was predominantly expressed intracellularly (supplemental Fig. S7). A higher concentration of furin inhibitor (50 μM) enhanced this effect and reduced PCSK9_55 levels in the extracellular compartment (supplemental Fig. S8).

It should be noted that purified furin is able to induce cleavage of PCSK9_62 from extracts of HEK293T cells overexpressing PCSK9 (supplemental Fig. S9).

**Fig. 3:**
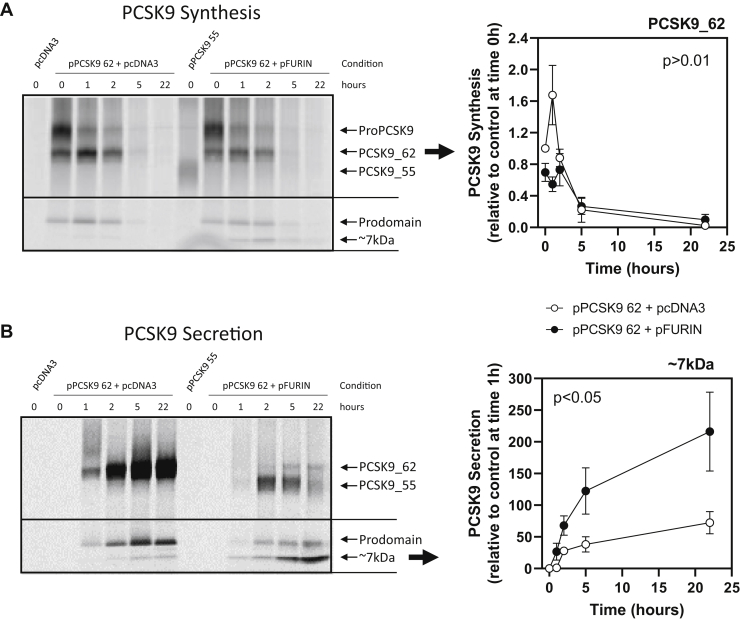
Kinetics of PCSK9_62 conversion to PCSK9_55 in vitro. Pulse-chase experiments traced the rate of PCSK9 production intracellularly and the rate of secretion to the extracellular space. Storage phosphor screen scans show PCSK9 presence over time (0–22 h) in cell extracts (A) and media (B) of HEK293T cells cotransfected with pPCSK9 62 and empty vector (empty circles) or pPCSK9 62 and pFURIN (full circles). Quantification of PCSK9_62 bands from “A” are plotted relative to pPCSK9 62 and empty vector condition at time 0 h, whereas for “B”, the 7 kDa peptide bands are plotted relative to pPCSK9 62 condition and empty vector at time 1 h. Image is a representative of 3 independent experiments. Values are mean ± SEM. Cells transfected with pcDNA3 were used as a negative control, and pPCSK9 55 was used as a reference to identify intracellular PCSK9_55.

Kinetic changes of PCSK9 production and secretion upon furin cleavage were studied using a pulse-chase approach (Fig. 3). Pulse-chase experiments corroborated the appearance of PCSK9_55 only at the extracellular space. The presence of furin decreased total PCSK9, as previously seen in the Western blots. The decrease in PCSK9_62 was seen intracellularly during the first hour of chasing, 67 ± 3% less PCSK9_62 in the presence of furin (*P* < 0.01) (Fig. 3A), and later in the secreted pool (overall, 86 ± 3% less PCSK9_62, *P* < 0.001) (Fig. 3B). Along with the decrease in PCSK9_62, there was a decrease in the prodomain levels, both intracellularly and extracellularly (55 ± 15% and 69 ± 10% less prodomain after 1 h of chasing, respectively [both *P* < 0.001]). Furin also induced the appearance of the ∼7 kDa peptide cleaved from PCSK9_62 to generate PCSK9_55. The released peptide was detected after 1 h of chasing intracellularly and extracellularly. Despite detecting the cleaved ∼7 kDa peptide intracellularly, PCSK9_55 was only detected in the extracellular compartment (Fig. 3A, B). Contrary to PCSK9_62 and the ∼7 kDa peptide kinetics that accumulate over time in the extracellular compartment, PCSK9_55 levels increased during the earlier time points, with a maximum of 607 ± 219% after 2 h of chasing (*P* < 0.07), but then decayed (Fig. 3B).

These data suggest that a pericellular furin pool is likely the major driver of PCSK9_55 formation in vivo. In addition, these data supports that furin induces a decrease in total PCSK9 by reducing PCSK9_62 levels and generating a product, PCSK9_55, with a shorter half-life than PCSK9_62.

### PCSK9_55 is not secreted by cells

**Fig. 4:**
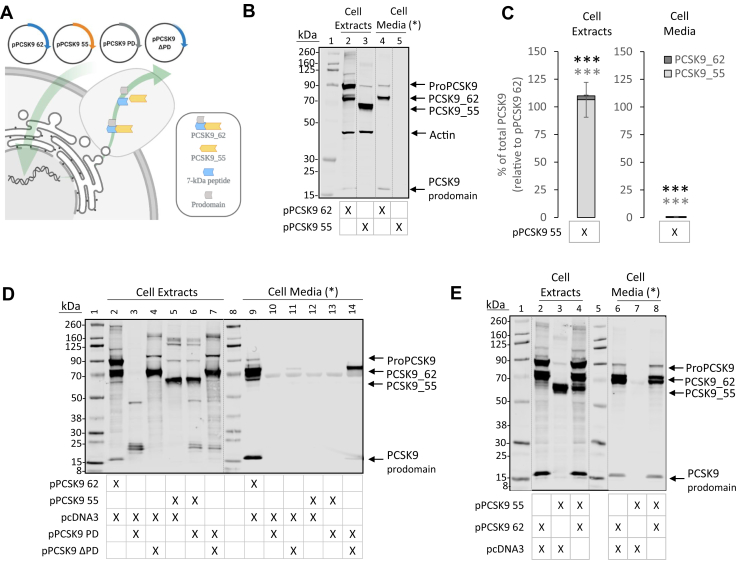
Intracellular PCSK9_55 fails to be secreted to the extracellular space. (A) Experimental strategy followed to characterize the engineered PCSK9_55 in cell culture. (B) Extracts and conditioned media of HEK293T cells transfected with pPCSK9 62 (lanes 2 and 4) or pPCSK9 55 (lanes 3 and 5) were separated via SDS-PAGE and immunoblotted for PCSK9 and actin (MW ladder; lane 1). Image is representative of ≥11 independent experiments. (C) Quantification of PCSK9 bands from “B” are plotted relative to pPCSK9 62 in the cell extracts and cell media. (D) Extracts and conditioned media of HEK293T cells transfected with pPCSK9 62 and empty vector (lanes 2 and 9), pPCSK9 PD and empty vector (lanes 3 and 10), pPCSK9 ΔPD and empty vector (lanes 4 and 11), pPCSK9 55 and empty vector (lanes 5 and 12), pPCSK9 55 and pPCSK9 PD (lanes 6 and 13), and pPCSK9 ΔPD and pPCSK9 PD (lanes 7 and 14) were separated via SDS-PAGE and immunoblotted for PCSK9 (MW ladder; lanes 1 and 8). Image is representative of 2 independent experiments. (E) Extracts and conditioned media of HEK293T cells transfected with pPCSK9 62 and empty vector (lanes 2 and 6), pPCSK9 55 and empty vector (lanes 3 and 7), and pPCSK9 62 and pPCSK9 55 (lanes 4 and 8) were separated via SDS-PAGE and immunoblotted for PCSK9 (MW ladder; lanes 1 and 5). Image is representative of 1 independent experiment. Values are mean ± SEM. ∗∗∗*P* < 0.001. (∗) Cell media is loaded with unequal protein load as in cell extracts. Dotted lines indicate splicing of the original image.

We engineered an expression vector to produce a recombinant form of PCSK9_55 (supplemental Fig. S10). HEK293T cells were transfected with either pPCSK9 62 or pPCSK9 55. Transfection with pPCSK9 62 led to the expression of intracellular ProPCSK9 and PCSK9_62 and to the secretion of PCSK9_62. In contrast, the transfection with pPCSK9 55 resulted in abundant cellular expression of PCSK9_55, which was entirely retained intracellularly (Fig. 4A–C). Digestion of cellular N-glycosylated PCSK9 with endoglycosidase H showed that PCSK9_55 was 2 times more sensitive than PCSK9_62 (*P* < 0.05) (supplemental Fig. S11), indicative of having higher levels of protein retained in the ER (15).

A PD-deficient PCSK9 will assemble *in trans* with an orthologous prodomain and will be efficiently secreted (21, 46, 47) (Fig. 4D). Our engineered pPCSK9_55 lacks the sequence that codes for the prodomain (supplemental Fig. S10). Cotransfection of pPCSK9_55 with pPCSK9 PD did not result in secretion of PCSK9_55 (Fig. 4D). Similarly, coexpression of pPCSK9 55 with a prodomain donor such as pPCSK9_62 did not promote the secretion of PCSK9_55 (Fig. 4E).

These results suggest that the intracellular pool of PCSK9_55 is retained in the cell owing to impaired transport in the secretory pathway and this retention cannot be resolved by the prodomain.

### Intracellular PCSK9_55 induces LDLR degradation

**Fig. 5:**
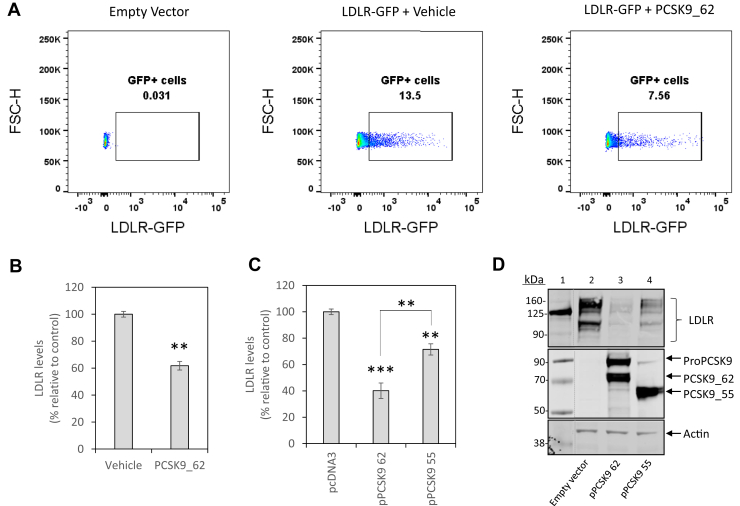
Development and validation of a functional assay to quantify PCSK9 activity on cellular LDLRs. LDLR levels in HEK293T cells are monitored by flow cytometry. (A) Representative plots that capture LDLR content as the percentage of GFP-positive cells (forward scatter height against GFP signal) in cells transfected with an empty vector (left plot), transfected with an LDLR-GFP-tagged expression vector and incubated with a vehicle (center) and transfected with an LDLR-GFP-tagged expression vector and incubated with a recombinant PCSK9_62 (3 μg/mL) for 24 h (right). (B) Quantification of LDLR levels upon incubation with recombinant PCSK9_62, as in “A.” (C) Quantification of LDLR levels upon transfection with pPCSK9 62 or pPCSK9 55. (D) Cell extracts (same conditions as in “C”) after separation by SDS-PAGE and immunoblotting for LDLR, PCSK9, and actin. Images are representative of 2 independent experiments. Data are representative of > 4 independent experiments that were run in duplicate or triplicate. Values are mean ± SEM. ∗∗*P* < 0.01 and ∗∗∗*P* < 0.001. Dotted lines indicate splicing of the original image.

To study whether intracellular PCSK9_55 maintains LDLR degradative activity relative to that of PCSK9_62, we developed a cell-based functional assay to monitor LDLR levels (see Methods and supplemental Fig. S1). To validate our assay, HEK293T cells overexpressing LDLR-GFP were incubated with recombinant PCSK9_62 (3 μg/mL for 24 h) (Fig. 5A). Incubation with recombinant PCSK9_62 reduced LDLR levels by 38 ± 3% (*P* < 0.01; Fig. 5B). To compare the PCSK9_62 function with that of PCSK9_55, we transfected HEK293T cells expressing LDLR-GFP with pPCSK9 62 or pPCSK9 55. Expression of PCSK9_62 reduced LDLR levels by 60 ± 6% (*P* < 0.001), whereas PCSK9_55 only reduced LDLR levels by 28 ± 4% (*P* < 0.01), significantly less than the full-length protein (*P* < 0.01) (Fig. 5C and supplemental Fig. S12). We validated these results using SDS-PAGE of cell extracts immunoblotted for LDLRs (Fig. 5D). To determine whether the intracellular accumulation of PCSK9_55 induced ER stress and activated the unfolded protein response as a nonspecific cause of LDLR degradation, we evaluated the expression of X-box binding protein-1 (XBP-1), a transcription factor and modulator of the unfolded protein response whose expression increases with ER stress (45), using qRT-PCR. No differences were observed in LDLR-expressing HEK293T cells transfected with either pPCSK9 62 or pPCSK9 55 (supplemental Fig. S13).

These data indicate that intracellular PCSK9_55 retains the ability to degrade LDLRs, but its activity is reduced by 50% compared with the mature form, PCSK9_62.

## Discussion

Our study contributes to the characterization of furin-cleaved PCSK9 by demonstrating that plasma PCSK9_55 is formed in the extracellular compartment and that the intracellular pool of PCSK9_55 cannot be secreted from the cell but is still capable of degrading LDLRs through an intracellular pathway. Our in vitro model shows that overexpression of PCSK9_62 and furin in cells generates PCSK9_55 in the extracellular space. Under these circumstances, inhibition of furin selectively impacted the extracellular PCSK9_55 pool. A pulse-chase approach confirmed that PCSK9_55 can only be detected at the extracellular space and suggests a pericellular site of action for furin to cleave PCSK9_62. Moreover, our studies show that the intracellular pool of PCSK9_55 generated by recombinant expression is retained intracellularly because it fails to assemble *in trans* with the orthologous prodomain that propels PCSK9 out of the cell. Finally, we demonstrate that PCSK9_55 is capable of degrading LDLRs through an intracellular pathway, although at 50% reduced activity when compared with PCSK9_62.

Our data strongly support the contention that plasma levels of furin-cleaved PCSK9 originate from the extracellular space. Previous work by Benjannet *et al.* discarded the role for extracellular furin in cleaving PCSK9_62 and proposed that furin localized in the transmembrane or in the Golgi generates PCSK9_55 (15, 22). In our hands, coexpression of PCSK9_62 and furin in both HEK293T and HepG2 cells generated PCSK9_55 that accumulated predominantly in the cell media. Furthermore, under the presence of a cell-permeable furin inhibitor, the conversion of PCSK9_62 to PCSK9_55 was predominantly affected within the extracellular space. Kinetic visualization of PCSK9_55 generation locates the cleaved form only at the extracellular space. These experiments confirm that furin cleaves PCSK9_62 in the extracellular space. We also observed that expressed and overexpressed furin mostly localized in the cellular compartment. Since recombinant furin is able to cleave PCSK9_62 in both cell extracts and cell media, we hypothesize that, in the living cell, PCSK9_62 is protected from furin by spatial compartmentalization, similarly to the observations of Genefra *et al.* (48). The pulse-chase experiments highlight the kinetics of the release of ∼7 kDa peptide from PCSK9_62 upon furin digestion. At the same time that PCSK9_55 can be detected extracellularly, the ∼7 kDa peptide can be detected intracellularly and extracellularly. Based on these observations, we propose that PCSK9_62 is cleaved by a pericellular furin pool. Aside from generating PCSK9_55, the coexpression of PCSK9_62 and furin reduced total PCSK9 protein. This was confirmed in the pulse-chase experiments showing less PCSK9_62 in the cell cotransfected with furin and decay in the generated PCSK9_55. Altogether, these data suggest that the PCSK9_55 form has a shorter half-life than its precursor PCSK9_62. Whether the half-lives of PCSK9 isoforms bound to lipoproteins are different remains to be determined.

Our experiments support the notion that intracellular PCSK9_55 is not secreted into the extracellular space, as transfection with a plasmid engineered to exclusively produce the PCSK9_55 form (pPCSK9 55) generated a protein that was only detected intracellularly. We tested the effect on secretion of coexpressing PCSK9 prodomain with prodomain-free PCSK9_62 or PCSK9_55. While cotransfection of the prodomain with the prodomain-free PCSK9_62 led to effective secretion of the protein, as expected (21, 46, 47), cotransfection of the prodomain with prodomain-free PCSK9_55 did not induce secretion. These results suggest that PCSK9_55 may lose the ability to bind the prodomain, ultimately leading to impaired secretion. Moreover, our Western blot analyses demonstrate that the extracellular PCSK9_55 product derived from pPCSK9 62 and pFURIN cotransfection is predominantly prodomain-free. Similarly, in our pulse-chase experiments, cotransfection of HEK293T cells with pPCSK9 62 and pFURIN results in the extracellular accumulation of PCSK9_55 and the unbound PCSK9 prodomain, demonstrating further that PCSK9_55 is not bound to the prodomain. These results are in concordance with our observations demonstrating that cotransfecting cells with pPCSK9 55 and pPCSK9 PD is unable to induce the secretion of PCSK9_55. These findings are in line with previous reports showing that PCSK9_55 loses the prodomain (15, 17, 24). Altogether, our findings, in combination with existing knowledge, support a scenario in which PCSK9_55 is generated in the extracellular compartment because once PCSK9 is cleaved by furin, the release of the prodomain prohibits the secretion of PCSK9 to the extracellular compartment. Thus, the small amount of intracellular PCSK9_55 detected upon cotransfection of pPCSK9 62 and pFURIN is likely to derive from hepatic uptake rather than intracellular production.

Changes in plasma furin levels may affect PCSK9 levels or function. Furin is a ubiquitous protein expressed in several cellular compartments including the trans-Golgi network, endosomes, and cell surface. In the ER, profurin's 83 amino acid prodomain facilitates its autocleavage where it remains bound to furin acting as a potent autoinhibitor during its transport to the trans-Golgi network/endosomes where the acidic pH promotes the autoproteolytic cleavage of the prodomain from furin, thus activating the enzyme (28). A transmembrane pool can undergo shedding and give rise to the circulating protein. Furin processes multiple targets (i.e., PCSK9, insulin receptor, insulin-like growth factor-1, β-nerve growth factor, Notch, β-amyloid precursor protein, and more) in multiple locations. Within the plasma compartment, LDL, but not VLDL, antagonizes furin's ability to cleave PCSK9 (26), suggesting that the protease preferentially acts on free PCSK9. Additional studies are required to determine whether LDL interferes with processing of other furin targets. Gene variants of furin have been associated with metabolic outcomes. In a case report, a point mutation of furin at the insulin proreceptor-processing site was associated with diabetes (49). Additionally, a furin polymorphism was associated with decreased triglyceride and increased HDL-C levels (50). Finally, elevated circulating furin levels were found to be associated with the metabolic syndrome (51), and a furin variant was linked to a higher risk of developing coronary artery disease (52).

It should be mentioned that, in humans, the ratio between PCSK9 and furin in plasma varies over 2 orders of magnitude (53, 54). The true physiological PCSK9-to-furin ratio in the cellular microenvironments where interaction between the 2 may occur is not known. In our in vitro proteolysis experiments, we chose a PCSK9-to-furin ratio of 2.8 to achieve roughly 80% efficiency of PCSK9_62-to-PCSK9_55 conversion.

Our data demonstrate that PCSK9_55 induces the degradation of LDLRs through an intracellular pathway. Several in vitro studies demonstrate that extracellular PCSK9_55 has reduced or null LDLR degradation activity compared with PCSK9_62 (15, 24, 25). Although it is known that PCSK9_62 induces LDLR degradation in both extracellular and intracellular pathways (35, 36), it was not known whether this would also be the case for PCSK9_55. To determine the functional role of PCSK9_55, we developed a simple method to quantify PCSK9-induced LDLR degradation. To assess PCSK9 activity, we utilized a cell-based assay where LDLRs tagged with GFP are overexpressed in cell culture and can be traced directly by flow cytometry. This strategy is less cumbersome than previously used methods that combine immunolabeling and flow cytometry (25, 55) because it does not require antibodies, thus reducing cost and likelihood of artifactual results. Using this cell-based flow cytometry approach, we report that intracellular PCSK9_55 degrades the LDLR at 50% capacity relative to PCSK9_62. However, we did not investigate the effect of extracellular PCSK9_55 on LDLRs, and thus, our results do not directly contradict the findings of Lipari *et al*. (25). Our previous work demonstrated that LDL-bound PCSK9 has a stronger binding affinity for the EGF-A domain of the LDLR than unbound PCSK9 (26). This finding suggests that apoB-bound PCSK9 is the more active plasma compartment. However, other reports contradict this hypothesis by showing that coincubation with LDL decreases LDLR degradation and hepatic uptake of PCSK9.

**Fig. 6:**
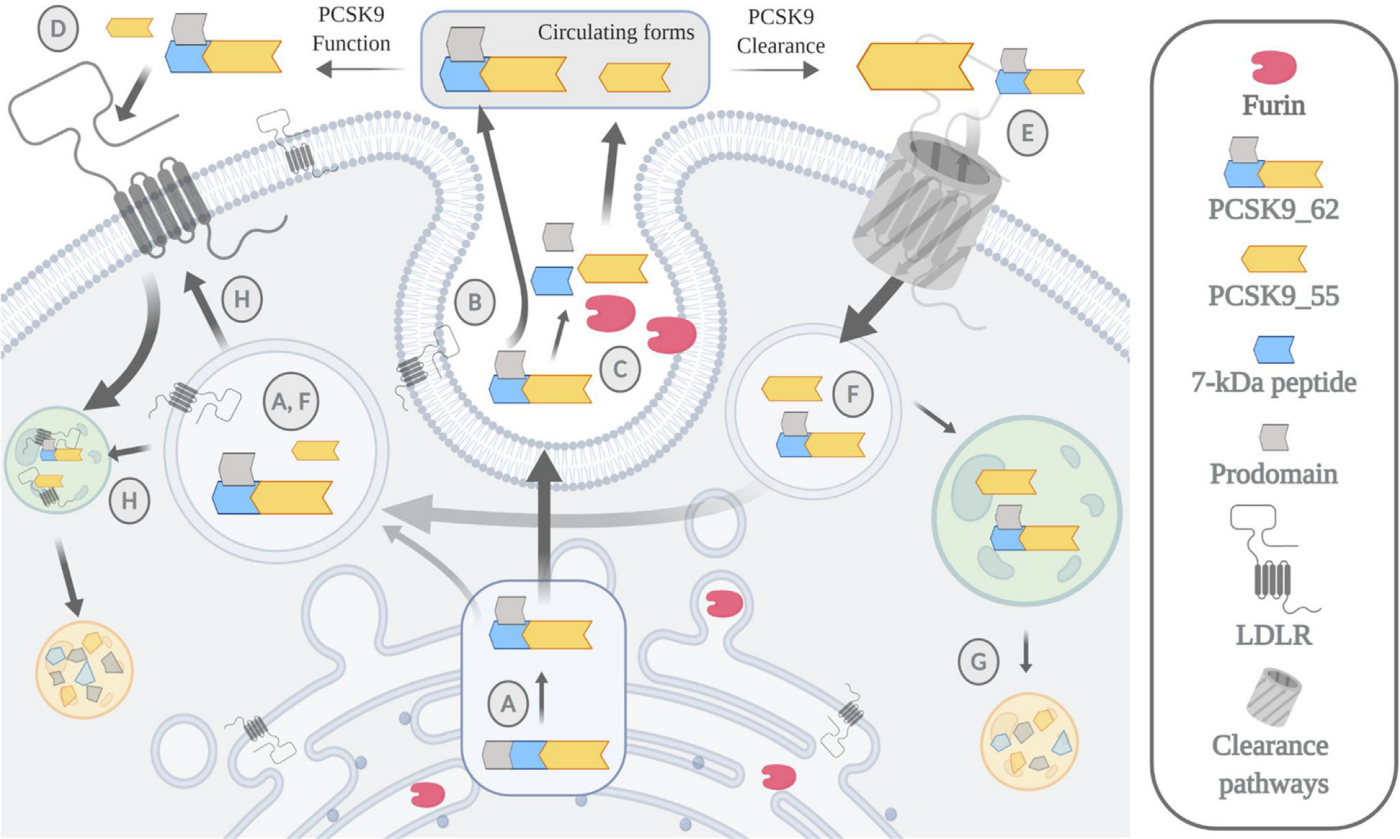
Working model. Newly synthesized PCSK9 undergoes an autocatalytic cleavage inside the cell that releases the prodomain (13 kDa) from the peptide chain. (A) The cleaved prodomain binds back to the main protein through noncovalent forces generating a heterodimer of 62 + 13 kDa, representing the mature form of PCSK9 (PCSK9_62). (B) This is an essential step for the proper secretion of PCSK9_62 into circulation. PCSK9_62 can also undergo a second cleavage, in the extracellular space, mediated by the protease furin. (C) Furin cleaves PCSK9 at the N-terminal region releasing an ∼7 kDa peptide and potentially the prodomain, generating the second most common form of plasma PCSK9 with a size of 55 kDa (PCSK9_55). (D) Once in circulation, both forms of PCSK9 induce hepatic LDLR degradation, though PCSK9_62 is more active than PCSK9_55. (E) PCSK9 elimination from circulation is mediated by LDLR-dependent and LDLR-independent pathways. Potentially, the furin-cleaved form of PCSK9, PCSK9_55, has a shorter half-life owing to a faster clearance rate than PCSK9_62. (F) Once inside the cell, PCSK9_55 cannot get secreted back to circulation and (G) before going through catabolism, (H) the intracellular pool of PCSK9_55 is capable of inducing LDLR degradation though less so relative to PCSK9_62.

Herein, we propose a model where furin acts as a metabolic deactivator of PCSK9 (Fig. 6). Our data suggest that furin cleaves PCSK9 extracellularly, inducing the release of a 7 kDa peptide and the detachment of the prodomain. This posttranslational modification reduces PCSK9's ability to degrade the LDLR through the extracellular pathway. Our data suggest that the plasma clearance of PCSK9_55 is faster than that for the mature form. Once internalized, PCSK9_55 cannot be resecreted owing to the lack of the prodomain. This retained PCSK9_55 pool is capable of inducing LDLR degradation through and intracellular pathway.

In summary, we show that plasma PCSK9_55 is generated by the extracellular action of furin, that PCSK9_55 has a shorter half-life than PCSK9_62, and that intracellular PCSK9_55 cannot be secreted but retains LDLR degradation activity and therefore the ability to influence cholesterol homeostasis.

### Data availability

Mass spectrometry raw files are available at PanoramaWeb (https://panoramaweb.org/kqwUPs.url). Additional raw data are available upon request.

## Conflict of interest

The authors declare that they have no conflicts of interest with the contents of this article
