# Supplementary material for: Insights into the kinetics and dynamics of the furin-cleaved form of PCSK9
Source: J Lipid Res. 2020 Nov 23;62:100003. doi: 10.1194/jlr.RA120000964 (PMC7890205; doi:10.1194/jlr.RA120000964)
Supplement: Supplemental Figures and Tables [file mmc1.pdf]

Insights into the kinetics and dynamics of the furin-cleaved form of PCSK9

Carlota Oleaga[1], Joshua Hay[1], Emma Gurcan[1], Larry L. David[2], Paul A. Mueller[1], Hagai Tavori[1], Michael D. Shapiro[1], Nathalie Pamir[1,*], Sergio Fazio[1]

[1]Knight Cardiovascular Institute, Center for Preventive Cardiology, and [2]Proteomics Shared Resource, Oregon Health & Science University, Portland, OR, USA

* Corresponding author: Nathalie Pamir, Knight Cardiovascular Institute, Center for Preventive Cardiology, Oregon Health & Science University, 3181 SW Sam Jackson Park Road, Mail Code HRC5N, Portland, OR 97239, USA. E-mail: pamir@ohsu.edu

Dr. Shapiro's current address is Center for Preventive Cardiology, Wake Forest University School of Medicine, Winston-Salem, NC 27157, USA.

Running title: Updates in furin-cleaved PCSK9 characterization

Supplemental Information

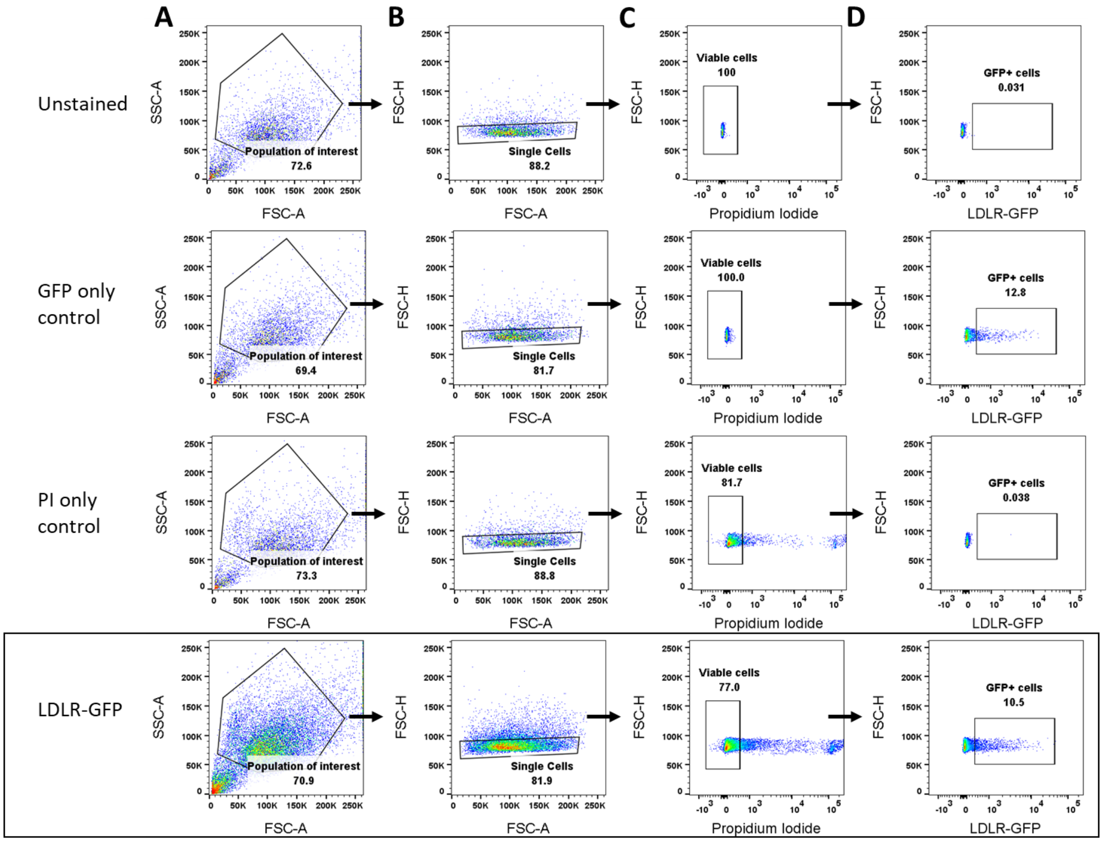

Figure S1. Gating strategy followed in PCSK9 Functional Assay. (A) Initially, the population of interest is gated out of the side and forward scatter plot, **(B)** followed by the selection of the single cell population, **(C)** then, live cells are gated by being Propidium iodide (PI) negative, and **(D)** finally, the LDLR-GFP positive populations are gated by expression of GFP. Representative plots of HEK293T cells analyzed by this protocol include compensation controls (5000 cells analyzed in unstained, GFP only control and PI only control) and LDLR-GFP transfected cells and stained for PI (10000 cells analyzed). Quantifications included in plots are percentage relative to the parental gate.

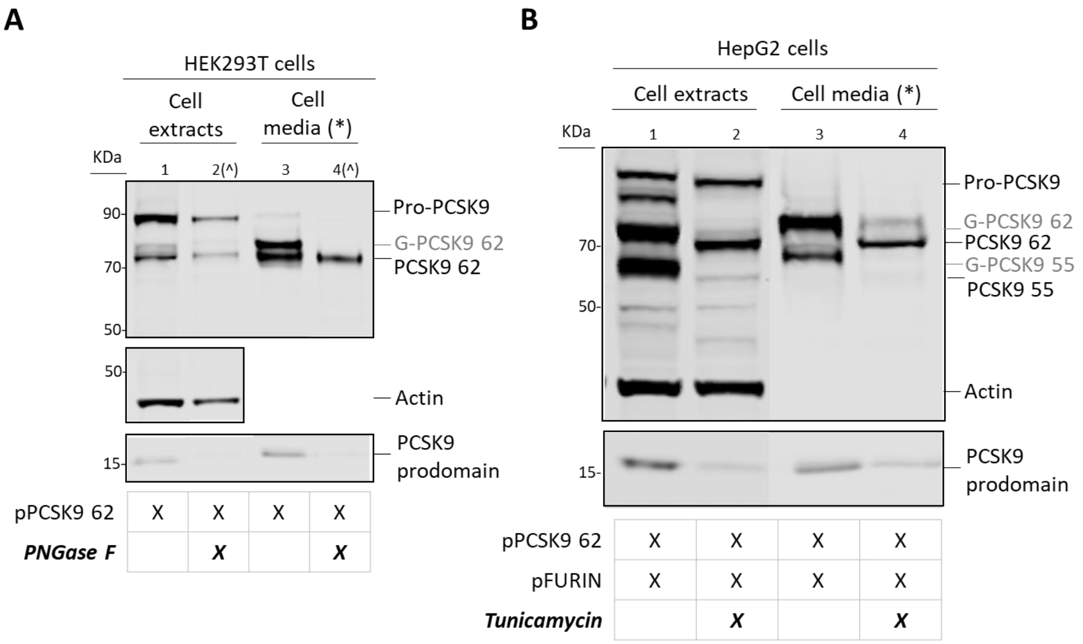

Figure S2. N-glycosylated forms of PCSK9. (A) Extracts and conditioned media of HEK293T cells transfected with pPCSK9 62 (lanes 1 and 3), and same extracts (^ only 20µg) pre-treated with PNGase F (lanes 2 and 4) were separated via SDS-PAGE and immunoblotted for PCSK9 and actin. Image is representative of 3 independent experiments. **(B)** Extracts and conditioned media of HepG2 cells co-transfected with pPCSK9 62 and pFURIN (lanes 1 and 3), and same conditions also treated with tunicamycin (lanes 2 and 4) were separated via SDS-PAGE and immunoblotted for PCSK9 and actin. Image is representative of 2 independent experiments. (*) Cell media is loaded with unequal protein load as in cell extracts.

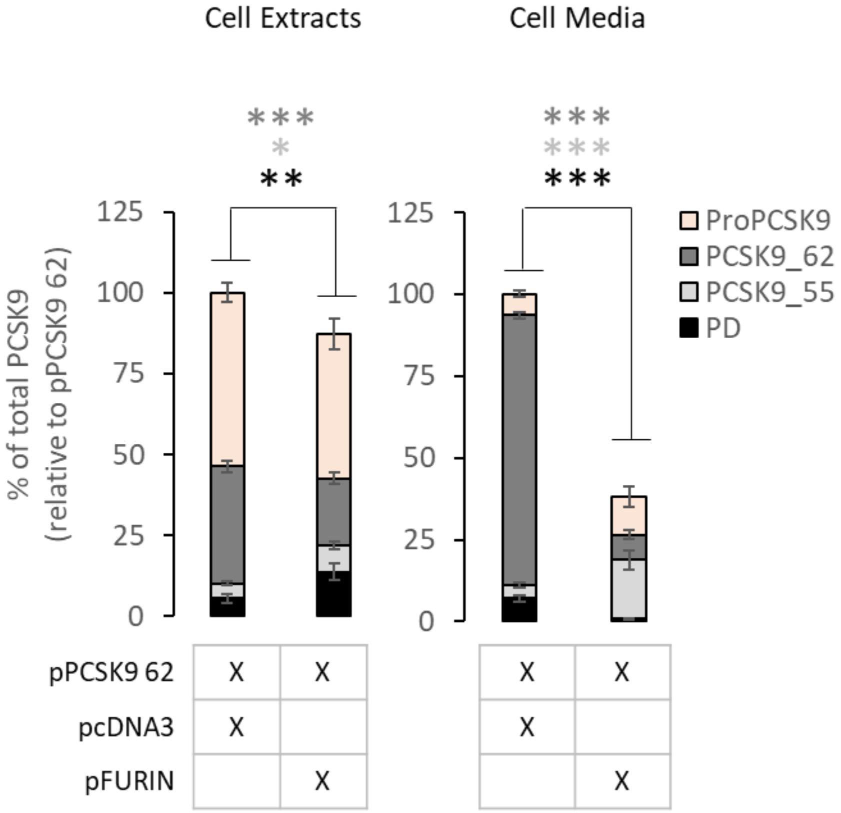

Figure S3. Changes in PCSK9 forms induced by furin co-transfection. Total PCSK9 (ProPCSK9, PCSK9_62, PCSK9_55 and PCSK9 prodomain bands) quantified in the intracellular and extracellular compartments of HEK293T transfected with pPCSK9 62 and empty vector or pPCSK9 62 and pFURIN. Data is representative of ≥13 independent experiments. Values are mean ± SEM. * $p<0.05$, ** $p<0.01$ and *** $p<0.001$.

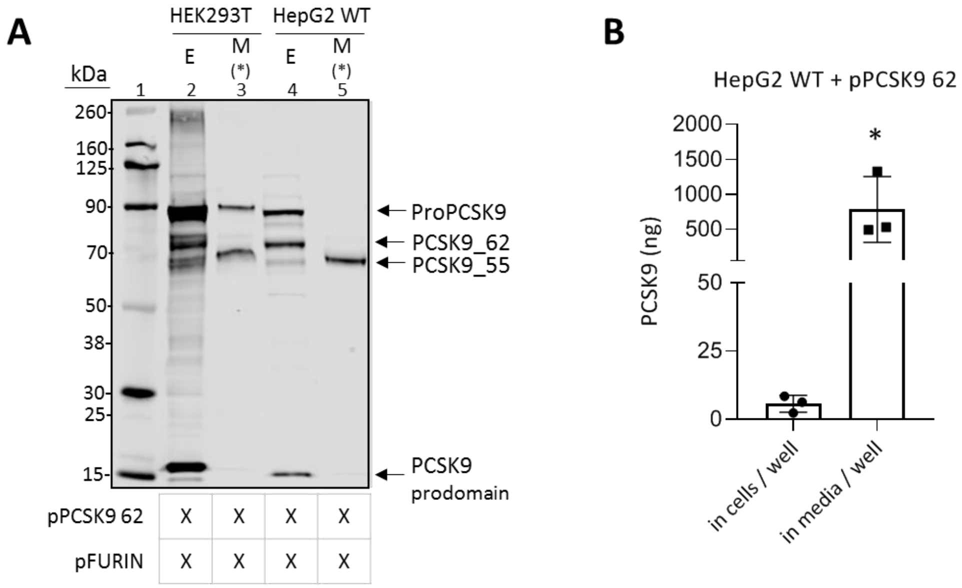

Figure S4. Additions to generation of PCSK9_55 in cells culture. (A) Extracts (E) and conditioned media (M) of HEK293T (lanes 2 and 3) and HepG2 (lanes 4 and 5) cells transfected with pPCSK9 62 and pFURIN were separated via SDS-PAGE and immunoblotted for PCSK9 (MW Ladder; Lane 1). Image is representative of 3 independent experiments. **(B)** Total PCSK9 quantified in the intracellular and extracellular compartments of our *in vitro* conditions in HepG2 cells transfected with pPCSK9 62. Data is representative of 3 independent experiment. Values are mean ± SEM. * $p<0.05$. (*) Cell media is loaded with unequal protein load as in cell extracts.

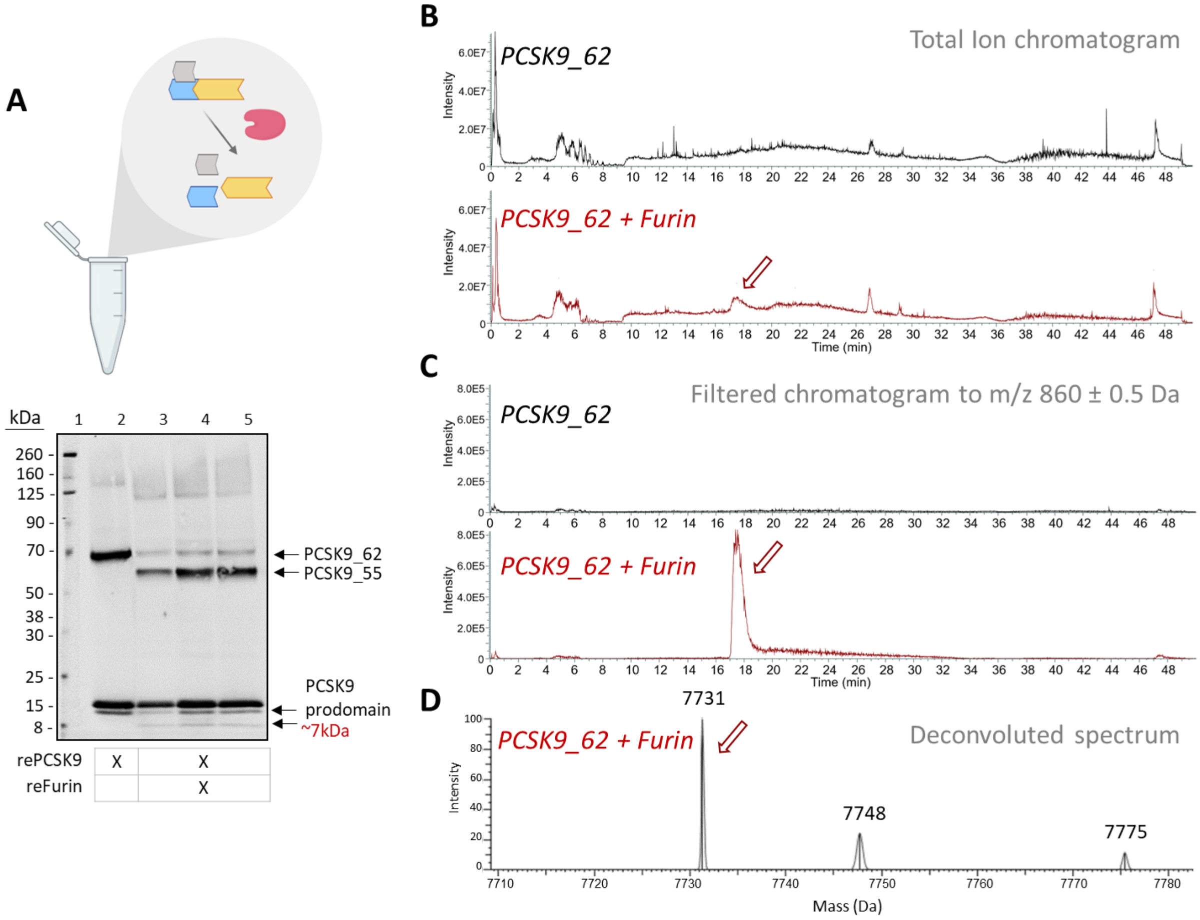

Figure S5. Furin cleavage specificity for PCSK9. (A) Purified recombinant PCSK9 and furin were incubated *in vitro* and PCSK9_55 production was evaluated. The reaction products from the *in vitro* incubations of purified recombinant PCSK9 without furin (lanes 1) and with furin (lanes 3-5) were separated via SDS-PAGE and immunoblotted for PCSK9. Image is representative of 9 independent experiments. **(B-D)** The reaction products from *in vitro* incubations of purified recombinant PCSK9 with, or without, furin (from "A") were assessed by LC-MS: (B) Total ion chromatograms and (C) chromatograms for ions with m/z= 859.9, corresponding to the major +9 charge state of the average mass of PCSK9 residues 153-218. The expected PCSK9 cleavage product (residues 153-218) was only observed in the furin cleaved sample (arrow). (D) The averaged spectra from the peak at 17.5 min in the furin cleaved sample was deconvoluted to yield a major species with mass = 7731, which was within the

instrumental error of the calculated average mass of 7730.5 for PCSK9 residues 153-218. The small higher molecular weight peaks at 7748 and 7775 were likely due to oxidation of methionine and carbamoylation of lysines, respectively. Carbamoylation was likely due to the present of urea during sample preparation.

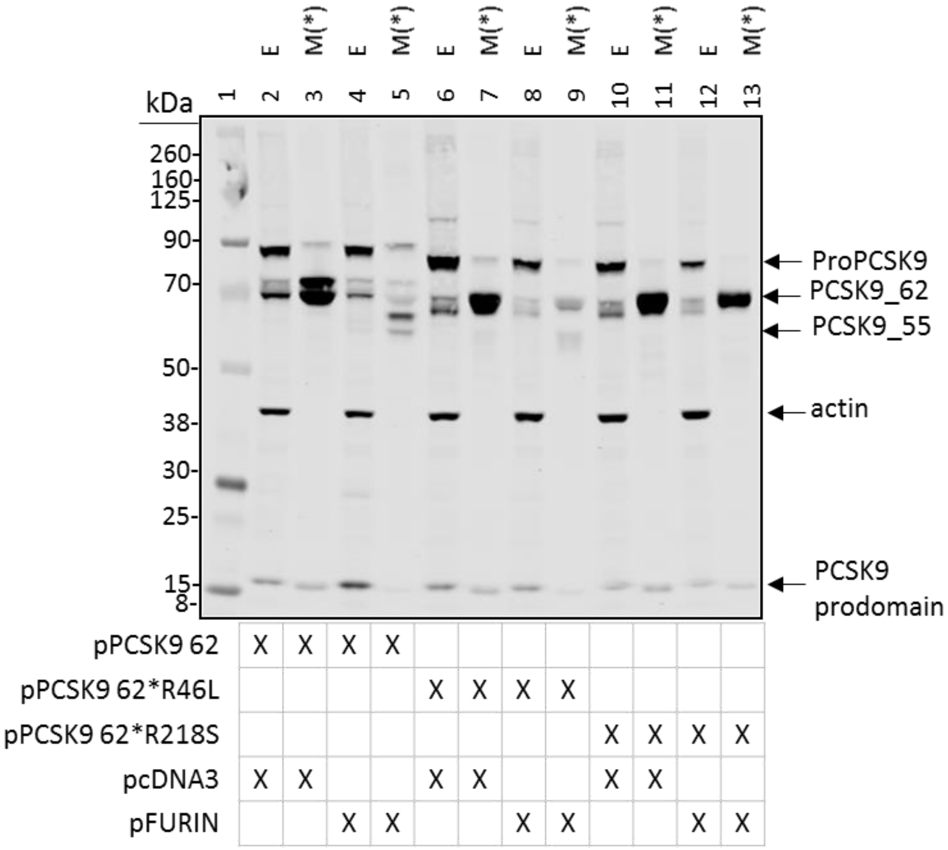

Figure S6. Furin cleavage is specific for PCSK9 in cell culture. Extracts (E) and conditioned media (M) of HEK293T cells co-transfected with pPCSK9 62 and empty vector (lanes 2-3), pPCSK9 62 and pFURIN (lanes 4-5), pPCSK9 62*R46L and empty vector (lanes 6-7), pPCSK9 62*R46L and pFURIN (lanes 8-9), pPCSK9 62*R218S and empty vector (lanes 10-11), and pPCSK9 62*R218S and pFURIN (lanes 12-13) were separated via SDS-PAGE and immunoblotted for PCSK9 and actin (MW Ladder;

Lane 1). Image is representative of 3 independent experiments. (*) Cell media is loaded with unequal protein load as in cell extracts.

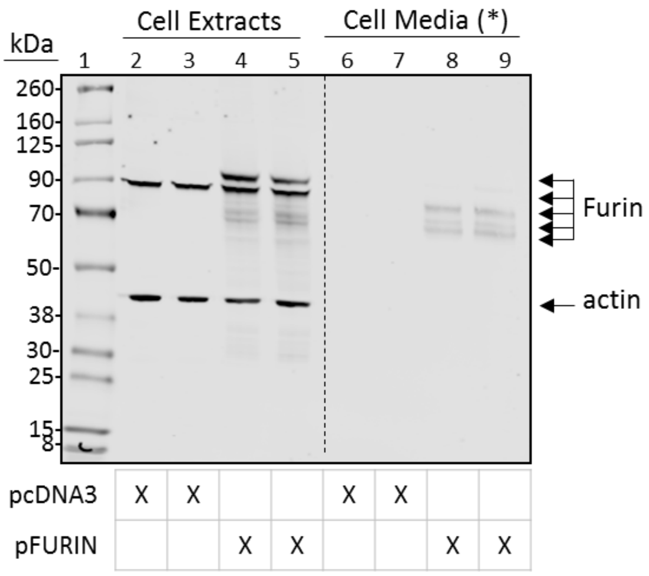

Figure S7. Furin expression in HEK293T cells. Extracts and conditioned media of HEK293T cells transfected with empty vector (lanes 2-3 and 6-7) or pFURIN (lanes 4-5 and 8-9) were separated via SDS-PAGE and immunoblotted for furin and actin. Image is representative of 3 independent experiments. (*) Cell media is loaded with unequal protein load as in cell extracts.

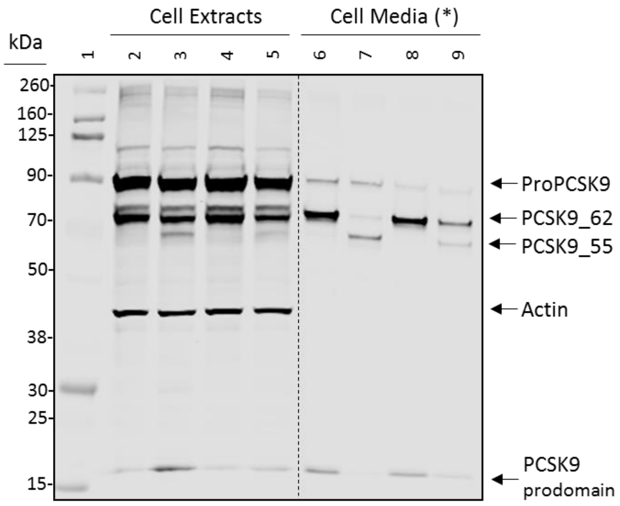

pPCSK9 62	X	X	X	X		X	X	X	X
pcDNA3	X		X		X	X		X	
pFURIN		X		X			X		X
dec-RVKR-cmk (50µM)			X	X				X	X

Figure S8. Furin inhibition in the generation of PCSK9_55. HEK293T cells co-transfected with pPCSK9 62 and pFURIN were treated with the cell-permeable furin inhibitor dec-RVKR-cmk (50µM) to repress PCSK9 cleavage. Extracts and conditioned media of HEK293T cells transfected with pPCSK9 62 and empty vector (lanes 2 and 6, and treated with furin inhibitor lanes 4 and 8) or pPCSK9 62 and pFURIN (lanes 3 and 7, and treated with furin inhibitor lanes 5 and 9) were separated via SDS-PAGE and immunoblotted for PCSK9 and actin (MW Ladder; Lane 1). Image is a representative of 1 independent experiment. (*) Cell media is loaded with unequal protein load as in cell extracts.

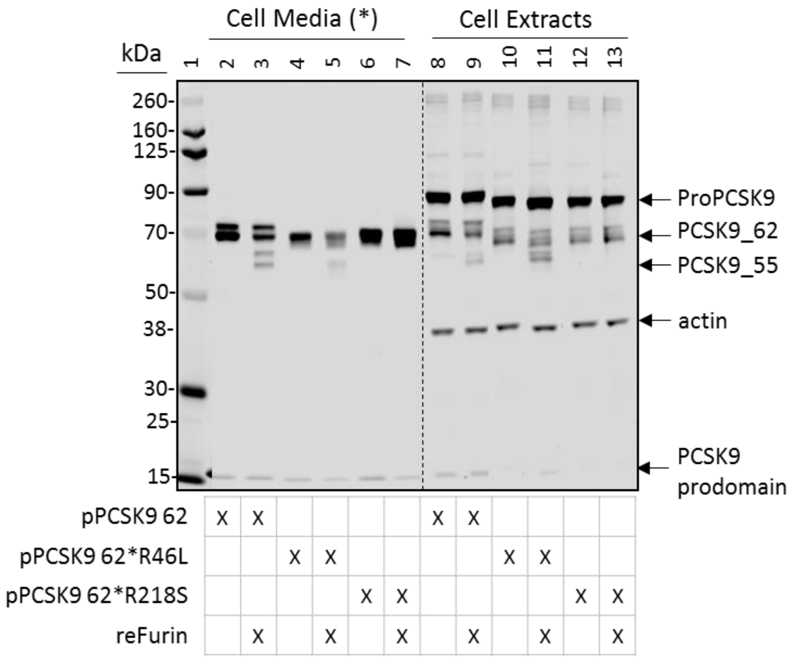

Figure S9. Additions to furin cleavage specificity for PCSK9. Extracts and conditioned media of HEK293T cells transfected with pPCSK9 62 (WT, R46L or R218S) were treated with a vehicle (lanes 2, 4, 6, 8, 10, 12) or a recombinant furin (lanes 3, 5, 7, 9, 11, 13). Reactions were separated via SDS-PAGE and immunoblotted for PCSK9 and actin (MW Ladder; Lane 1). Image is a representative of 2 independent experiments. (*) Cell media is loaded with unequal protein load as in cell extracts.

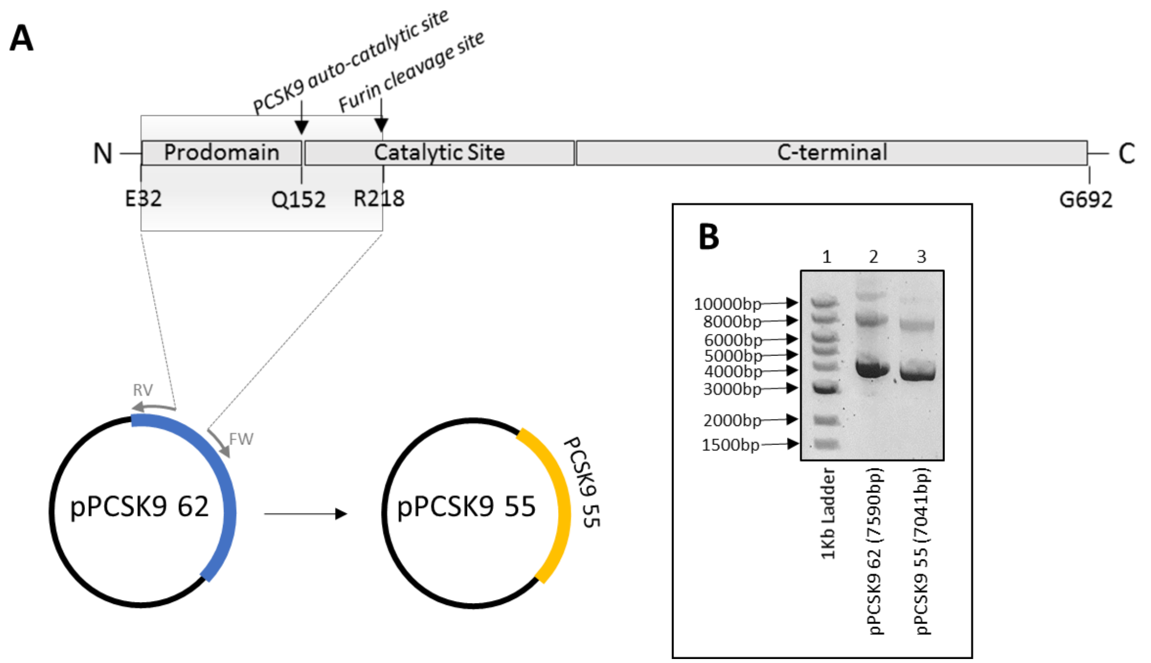

Figure S10. Generation of recombinant PCSK9_55. (A) Schema highlighting the strategy to generate pPCSK9 55. **(B)** DNA electrophoresis on agar gel showing plasmid size reduction from pPCSK9 62 to pPCSK9 55.

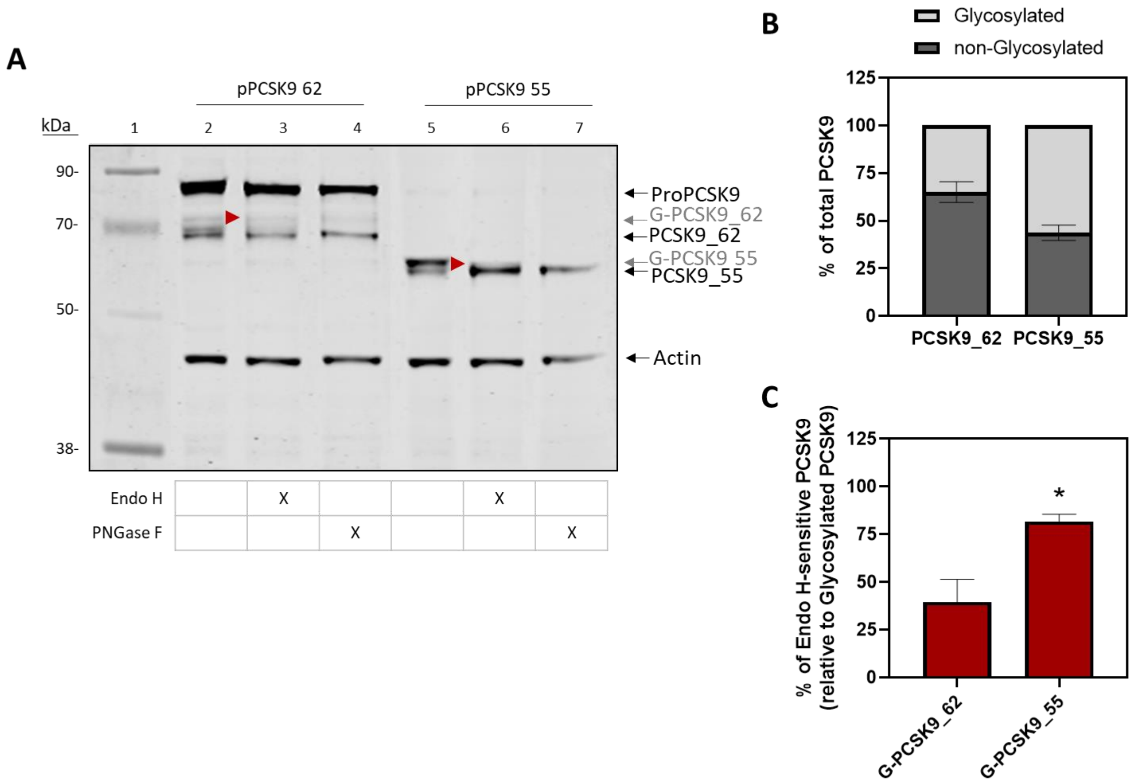

Figure S11. PCSK9 sensitivity to Endoglycosidase H digestion. (A) Extracts of HEK293T cells transfected with pPCSK9 62 (lanes 2-4) or pPCSK9 55 (lanes 5-7) were subjected to Endoglycosidase H or PNGase F digestion and separated via SDS-PAGE and immunoblotted for PCSK9 and actin (MW Ladder; Lane 1). Image is representative of 4 independent experiments. Arrowheads (red) identify the Endo H-resistant pools. **(B)** Quantification of the N-Glycosylated versus non-Glycosylated forms. **(C)** Quantification of the Endo H-sensitive pools of PCSK9. Data is representative of 4 independent experiment. Values are mean ± SEM. * $p<0.05$.

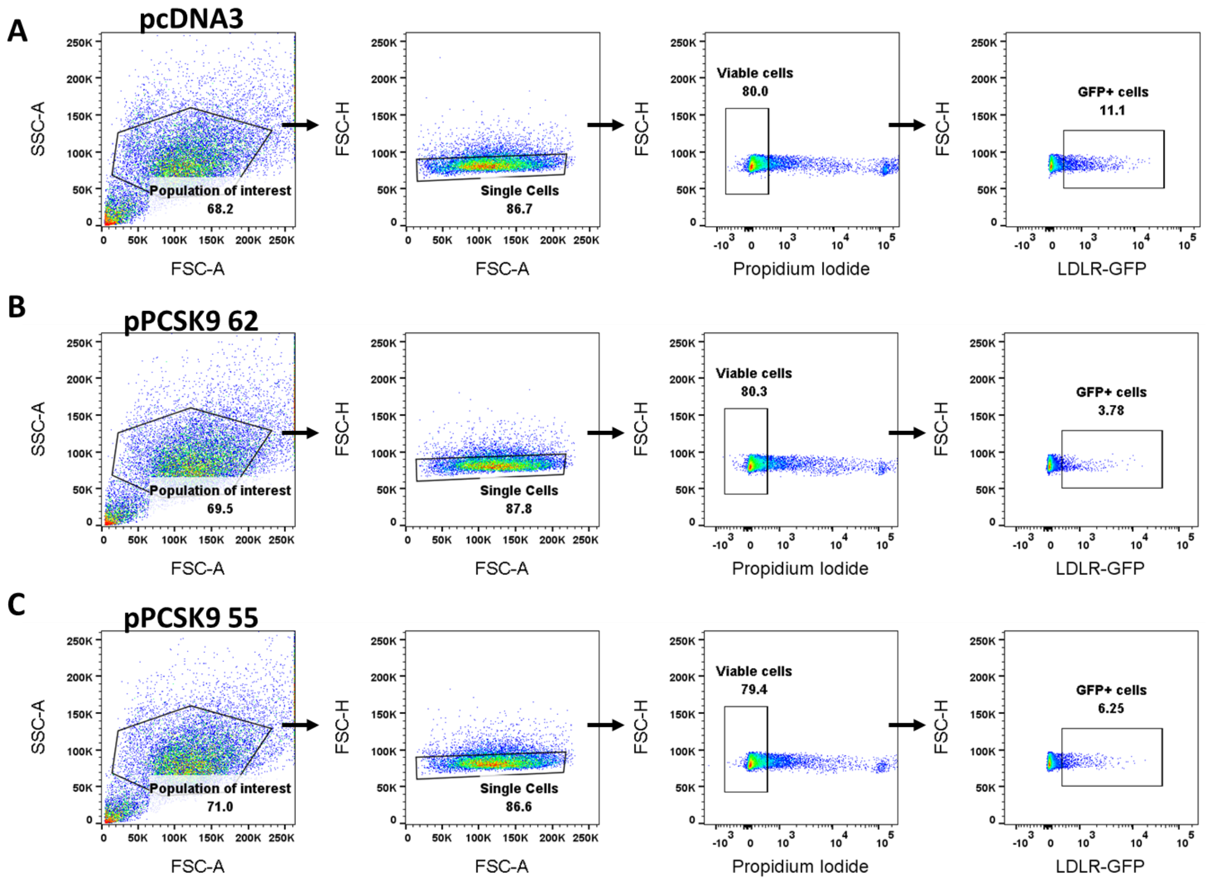

Figure S12. Comparison of PCSK9_62 and PCSK9_55 activity. Representative plots that capture LDLR content as percentage of GFP positive cells in HEK293T cells upon **(A)** transfection with empty vector, **(B)** pPCSK9 62 or **(C)** pPCSK9 55. Quantifications are plotted in Fig. 5C of the manuscript.

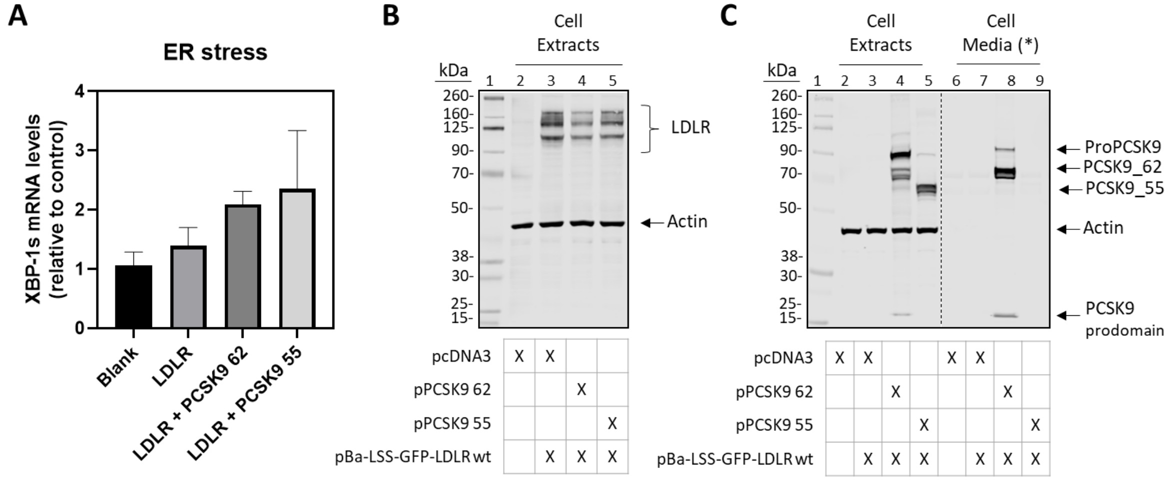

Figure S13. ER stress evaluation. (A) XBP-1s mRNA expression levels of HEK293T cells co-transfected with combinations of empty vector, pBa-LSS-GFP-LDLR wt, pPCSK9 62 and pPCSK9 55 are normalized to housekeeping RPL32 levels and plotted relative to control. Data is representative of 3 independent experiment. Protein extracts and conditioned media of HEK293T cells treated equally to the wells used to collect RNA, were separated via SDS-PAGE and immunoblotted for LDLR and actin **(B)** or PCSK9 and actin **(C)** (MW Ladder; Lane 1). Images are representative of 3 independent experiments. (*) Cell media is loaded with unequal protein load as in cell extracts. Values are mean ± SEM.

Table S1. Cell culture media.

Cell culture media	Item	Stock	Working concentration
HepG2	Sodium pyruvate (Cat# 11360070, Thermo Fisher Scientific)	100mM	1mM
	Penicillin/streptomycin (Cat# 15-140-122, Thermo Fisher Scientific)	1000X	1X
	FBS (Cat# 16000044, Thermo Fisher Scientific)	100%	10%
	DMEM (Cat# 11965-118, Thermo Fisher Scientific)		1X
HEK293T	Penicillin/streptomycin (Cat# 15-140-122, Thermo Fisher Scientific)	1000X	1X
	FBS (Cat# 16000044, Thermo Fisher Scientific)	100%	10%
	DMEM (Cat# 11885-084, Thermo Fisher Scientific)		1X

Table S2. DNA primer list.

Project	Product	Primer name	5’ - 3’
Inverse PCR	pPCSK9 55	FW-Δ32-218	CAGGCCAGCAAGTGTGACAGTCATGGCACC
		RV-Δ32-218	CTGCGCACGGGCGCCCGC
	pPCSK9 PD	FW-Δ152-692	AAGGGTCAAGACAATTCTGCAG
		RV-Δ152-692	CTGGGCAAAGACAGAGGAG

Table S3. Primary and secondary antibody list.

Primary Antibody	Cat#, Vendor	Dilution	Secondary Antibody	Cat#, Vendor	Dilution
Rabbit anti-hPCSK9	CY-P1037, MBL International	1:1000	Goat anti-rabbit	925-32211, LI-COR Biosciences	1:15000
Rabbit anti-hFurin	Ab183495, Abcam	1:1000	Goat anti-rabbit	925-32211, LI-COR Biosciences	1:15000
Goat anti-hLDLR	AF2148, R&D	1:1000	Donkey anti-goat	925-32214, LI-COR Biosciences	1:15000
Mouse anti-hActin	A5441, Sigma-Aldrich	1:2000	Goat anti-mouse	926-68070, LI-COR Biosciences	1:15000

Table S4. Media used in Pulse-chase experiments.

Pulse-chase media	Items	Stock	Working concentration
**Starvation medium**	Sodium pyruvate (Cat# 11360070, Thermo Fisher Scientific)	100mM	1mM
	Penicillin/streptomycin (Cat# 15-140-122, Thermo Fisher Scientific)	1000X	1X
	Glutamine (Cat# GLL01, Caisson Labs)	200mM	2mM
	DMEM -Met/Cys (Cat# 21013024, Thermo Fisher Scientific)		1X
**Pulse medium**	35S-Met/Cys mix (Cat# NEG77200, Perkin-Elmer)	11mCi/mL	0.3mCi/mL
	Sodium pyruvate (Cat# 11360070, Thermo Fisher Scientific)	100mM	1mM
	Penicillin/streptomycin (Cat# 15-140-122, Thermo Fisher Scientific)	1000X	1X
	Glutamine (Cat# GLL01, Caisson Labs)	200mM	2mM
	DMEM -Met/Cys (Cat# 21013024, Thermo Fisher Scientific)		1X
**Chasing medium**	Methionine (Cat# M9625, Sigma-Aldrich)	250mM	2mM
	Cysteine (Cat# C1276, Sigma-Aldrich)	500mM	2mM
	Sodium pyruvate (Cat# 11360070, Thermo Fisher Scientific)	100mM	1mM
	Penicillin/streptomycin (Cat# 15-140-122, Thermo Fisher Scientific)	1000X	1X
	DMEM (Cat# 11965-118, Thermo Fisher Scientific)		1X

Table S5. Estimation of PCSK9_55 amounts in the HEK293T *in vitro* system.

		pPCSK9 62 + pcDNA3		pPCSK9 62 + pFURIN	
		Cell extracts	Cell Media	Cell extracts	Cell Media
62-kDa band	PCSK9 ng	11.10 ng	932.52 ng	Estimated: 6.59 ng	Estimated: 95.39 ng
	% of total PCSK9	87.6 %	95.8%	52 %	98 %
55-kDa band	PCSK9 ng	Estimated: 1.57 ng	Estimated: 40.88 ng	Estimated: 2.68 ng	Estimated: 258.9 ng
	% of total PCSK9	12.4 %	4.2 %	21.2 %	26.6 %

Running title: Updates in furin-cleaved PCSK9 characterization
